# Engineering Human Beige Adipose Tissue

**DOI:** 10.3389/fbioe.2022.906395

**Published:** 2022-07-01

**Authors:** Maria A. Gonzalez Porras, Katerina Stojkova, Francisca M. Acosta, Christopher R. Rathbone, Eric M. Brey

**Affiliations:** ^1^ Department of Biomedical Engineering and Chemical Engineering, University of Texas at San Antonio, San Antonio, TX, United States; ^2^ Institute of Regenerative Medicine, University of Texas at San Antonio, San Antonio, TX, United States; ^3^ Department of Biochemistry and Structural Biology, University of Texas Health Science Center, San Antonio, TX, United States

**Keywords:** beige adipocyte, microvascular fragments, hydrogel, microtissue, obesity

## Abstract

In this study, we described a method for generating functional, beige (thermogenic) adipose microtissues from human microvascular fragments (MVFs). The MVFs were isolated from adipose tissue acquired from adults over 50 years of age. The tissues express thermogenic gene markers and reproduce functions essential for the potential therapeutic impact of beige adipose tissues such as enhanced lipid metabolism and increased mitochondrial respiration. MVFs serve as a potential single, autologous source of cells that can be isolated from adult patients, induced to recreate functional aspects of beige adipose tissue and enable rapid vascularization post-transplantation. This approach has the potential to be used as an autologous therapy for metabolic diseases or as a model for the development of a personalized approach to high-throughput drug development/screening for adipose tissue.

## Introduction

Adipose tissue plays a critical role in regulating systemic metabolism. Aberrant adipose tissue function and associated comorbidities generate an economic burden of over $190 billion annually on the US healthcare system (Cawley and Meyerhoefer, 2012; Saklayen, 2018). Pharmacologic interventions and lifestyle changes have not proven to be broadly successful in addressing this complex medical challenge. White adipocytes store energy and are the primary parenchymal cells in most adipose tissues. However, brown adipocytes and inducible brown-like cells in white adipose tissue, referred to as beige or brite adipocytes (Spiegelman, 2013; Wu et al., 2013; Bi et al., 2014), possess the ability to dissipate energy as heat. The activation of beige or brown adipose tissues (BATs) may improve the systemic metabolism and could serve as a method for treating obesity and metabolic disease.

Perhaps the most transformative application of this concept is the implantation of BAT to replace or supplement white adipose tissues (WATs) (Blumenfeld et al., 2018; White et al., 2019). The continuous improvement in surgical techniques for transplanting small adipose units (fat grafting) (Khouri and Khouri, 2017) provides indication of the translational potential of this approach. However, the use of donor brown or beige tissues is not practical, primarily, due to limited availability. Tissue engineering has been proposed as an alternative approach for producing autologous beige or brown adipose tissues of sufficient volume to have a clinical impact (Tharp et al., 2015; Vaicik et al., 2015).

Clinical application of engineered beige or brown adipose tissues requires the identification of an autologous source of cells. Microvascular fragments (MVFs) are microvessel units that are enzymatically isolated in large amounts from adipose tissue (Frueh et al., 2017b). The isolated units are segments of arteriolar, capillary, and venular origin with an average length of 40–50 μm (Frueh et al., 2017a). Due to their intact vessel morphology and functionality, they can rapidly reassemble into new microvascular networks and have been explored extensively as a method for vascularizing engineered tissues (Nunes et al., 2010).

Recently, we and others have demonstrated that stem cells with adipogenic potential are resident within MVFs after isolation (Laschke and Menger, 2015; Acosta et al., 2020). However, it was unknown if MVFs could be used as a source for generating adipose microtissues with thermogenic characteristics. The use of hMVFs to develop thermogenic adipose tissue may offer distinct advantages over other cell sources, such as adipose-derived stem cells (ADSCs) that have been previously explored for engineering beige or brown adipose tissues (Tharp et al., 2015; Vaicik et al., 2015). The intact vascular units in MVFs may allow rapid vascularization, improving tissue incorporation into the host and increasing cell survival. In addition, the adipocyte progenitor cells in MVFs are isolated in their natural perivascular environment which may improve the behavior, function, and survival. Although the overall number of cells isolated with MVFs may be lower than traditional cell therapies (such as ADSCs), adipose tissue from a single donor, would be expected to provide a sufficient number of MVFs.

In this study, we described a method for generating functional, beige adipose microtissues from human MVFs. The MVFs were isolated from adipose tissue acquired from adults over 50 years of age. The tissues reproduce functions essential for the potential therapeutic impact of beige adipose tissues. MVFs serve as a potential single, autologous source of cells that can be isolated from adult patients, induced to recreate functional aspects of beige adipose tissue, and enable rapid vascularization post-transplantation. This approach has the potential to be used as an autologous therapy and would have a transformative impact on metabolic therapies.

## Materials and Methods

### Microvascular Fragment Culture

Human MVFs (hMVFs) (Advanced Solutions Life Sciences, LLC, Louisville, KY) isolated from three different patients (52-, 54-, and 56-year-old females) were used in the current study. hMVFs (20,000/ml) were thawed and resuspended in DMEM with 20 mg/mL of fibrinogen (Sigma-Aldrich, St. Louis, MO.) and then mixed with 10 U/mL thrombin (MilliporeSigma, St. Louis, MO.) to form fibrin scaffolds in 96-well culture plates. Hydrogel volumes were varied depending on the analysis performed (gene expression: 100 µL, OCR: 30 µL, and for all other analyses: 50 µL gels). All experimental conditions were performed in triplicate.

hMVFs were cultured in growth media (GM), white adipogenic media (WAM), or beige adipogenic media (BAM). GM consisted of Dulbecco’s modified Eagle’s medium (DMEM) with 20% (v/v) fetal bovine serum and 1% Pen-Strep. WAM consisted of a 4-day treatment with induction media: DMEM/F12 containing 20% fetal bovine serum, 1% Pen-Strep, 10 μg/mL insulin, 10 μM forskolin, 1 μM dexamethasone, and 125 μM indomethacin; followed by maintenance media: DMEM/F12, 20% fetal bovine serum, 1% Pen-Strep, and 10 μg/mL insulin for 10 days. BAM consisted of a 4-day treatment with induction media: DMEM/F12 containing 20% fetal bovine serum, 1% Pen-Strep, 10 μg/mL insulin, 10 μM forskolin, 1 μM dexamethasone, 1 µM rosiglitazone, and 120 nM thyroid hormone (T3); followed by maintenance media: DMEM/F12, 20% fetal bovine serum, 1% Pen-Strep, 10 μg/mL insulin, 10 µM forskolin, 1 µM rosiglitazone, and 120 nM T3 for 10 days. The media were changed every other day. Unless stated otherwise, reagents were purchased from Sigma-Aldrich, St. Louis, MO.

### RNA Isolation and Quantitative RT-PCR

RNA was isolated from fibrin scaffolds (100 µL) containing hMVFs (*n* = 4 individual hydrogels/group) and purified using a QIAGEN RNeasy Mini Kit (Valencia, CA), according to manufacturer guidelines. mRNA concentrations were measured using a Take3 microvolume plates (BioTek, Winooski, VT) and then normalized to 150 ng of mRNA for conversion to cDNA. The isolated RNA was converted to cDNA using the iScript cDNA synthesis kit (Bio-Rad, Hercules, CA). Real-time quantitative polymerase chain reaction (qPCR) was performed using a CFX96 Touch Real-Time PCR Detection System (Bio-Rad, Hercules, CA). All primers used to carry out the analysis were predesigned primers (Sigma-Aldrich, St. Louis, Mo). A measure of 10 µL of iTaq Universal SYBR Green Supermix (Bio-Rad, Hercules, CA) were used for each reaction. Fold expression levels were calculated using the 2^-∆∆Ct^ method, where the GM gels were designated as the calibrator group, and the GAPDH expression was used as the endogenous control.

### Lipolysis Assay

The amount of glycerol released by hMVFs was measured using the Lipolysis Colorimetric Assay Kit, according to manufacturer’s guidelines (BioVision, Milpitas, CA). Briefly, after 14 days of differentiation, gels were washed, and 100 nM of isoproterenol was added to half the wells for 3 h to stimulate lipolysis. Following stimulation, 50 µL of media was collected and added to 50 µL of the reaction mix provided by the manufacturer. After 1 h of incubation, absorbance was read at OD 570 nm. A standard curve was used to calculate the amount of glycerol released.

### Insulin Stimulated Glucose Uptake Assay

A glucose uptake analysis was performed using Glucose Uptake-Glo Assay (Promega, Madison, WI), following manufacturer’s guidelines. Briefly, hMVFs were cultured in DMEM without serum or glucose for 24 h prior to the assay. Then, insulin (1 mM) was added to the cells for 2 h, followed by the addition of 2-deoxyglucose (0.1 mM) for 1 h. Finally, a 2-deoxyglucose-6-phosphate (2DG6P) detection reagent was used to quantify the amount of glucose internalized by the cells. After 2 h of incubation, luminescence was measured with a spectrophotometer (Biotek, Winooski, VT).

### Confocal Imaging and Analysis

The hydrogel microtissues were fixed with 4% formaldehyde for 2 h at room temperature, permeabilized with 0.5% Triton-X for 20 min, blocked using 10% goat serum for 2 h, and incubated with rhodamine-labeled Griffonia (Bandeiraea) Simplicifolia Lectin I (GS-1; Vector Labs, Burlingame, CA, 1:100) for 24 h at 4°C to stain vasculature, with boron-dipyrromethene for 3 h (BODIPY; ThermoFisher, D3922, 1:100) to stain lipids, and with DAPI (ThermoFisher, R37606) to stain the nuclei. Samples were imaged with a Leica TCS SP8 confocal microscope (Buffalo Grove, IL) over 100-µm thickness/10 µm per section of the entire well. Confocal image stacks were processed and analyzed by Leica LASX software version 3.5.2 (Wetzlar, Germany). Quantification of lipid or the vessel well coverage percentage (total volume of lipid or vessel staining over the total volume of imaged hydrogel) was performed on 3D depictions of entire wells containing treated hMVFs using the Leica 3D analysis toolkit. Confocal images of BODIPY-stained samples were processed by ImageJ, and pixel values < 90 were removed as they were considered the background. The processed images were analyzed using a custom MATLAB (MathWorks, Natick, MA) script that first applied Otsu’s thresholding algorithm to create binary images, followed by individual lipids segmented from clusters. To reduce additional background noise and unsegmented lipid clusters, objects with an area <5 pixels, >150 pixels, or with an eccentricity >0.7 were removed (0 is a circle; 1 is a line segment). The area of each remaining lipid droplet and the number of droplets were then evaluated. The laser intensity, confocal aperture, and photomultiplier gain were kept constant across all samples.

### OCR Analysis

Hydrogels (30 µL) were seeded into XF96 V3 PS tissue culture microplates and cultured for 14 days. Before the assay, cells were placed in a 37°C incubator without CO_2_ for 45 min. A Seahorse XFe96 Flux Analyzer (Seahorse Bioscience) was used to analyze the cellular metabolic activity of hMVFs after differentiation. The XF Analyzer measures the oxygen consumption rate (OCR) of live cells in real-time. After basal measurements, oligomycin (1 mM) was added to the culture wells (at 42 min) to inhibit ATP synthase, reducing the contribution of ATP production and revealing proton leak in the electron transport chain (ETC, after correcting for non-mitochondrial oxygen consumption). Maximum respiration was induced by exposure to the ionophore carbonyl cyanide-ptrifluoromethoxyphenylhydrazone (FCCP, 250 nM) at 84 min. Finally, rotenone and antimycin A (2 mM/2 mM) were used at 126 min to inhibit the ETC enzymes upstream of oxygen consumption, thus eliminating all mitochondrial oxygen expenditure. The final OCR represents the background non-mitochondrial oxygen consumption of the cells. Subtracting the final OCR after rotenone/antimycin A treatment from the initial OCR before oligomycin treatment gives the basal mitochondrial metabolism or the mitochondrial OCR at a resting state [41]. Six measurements were taken prior to, and following, the application of each drug solution.

### Statistical Analysis

GraphPad Prism Software 7 (GraphPad Software, Inc., La Jolla, CA) was used to run one, or two-way analysis of variance (ANOVA) tests with Holm–Sidak’s multiple comparison analyses to determine differences between groups. Statistical significance was defined as *p* < 0.05. All results are presented as mean ± standard error of the mean (SEM).

## Results

### Human MVFs Can Be Induced To Form Beige And White Adipocytes in 3D Culture

**FIGURE 1:**
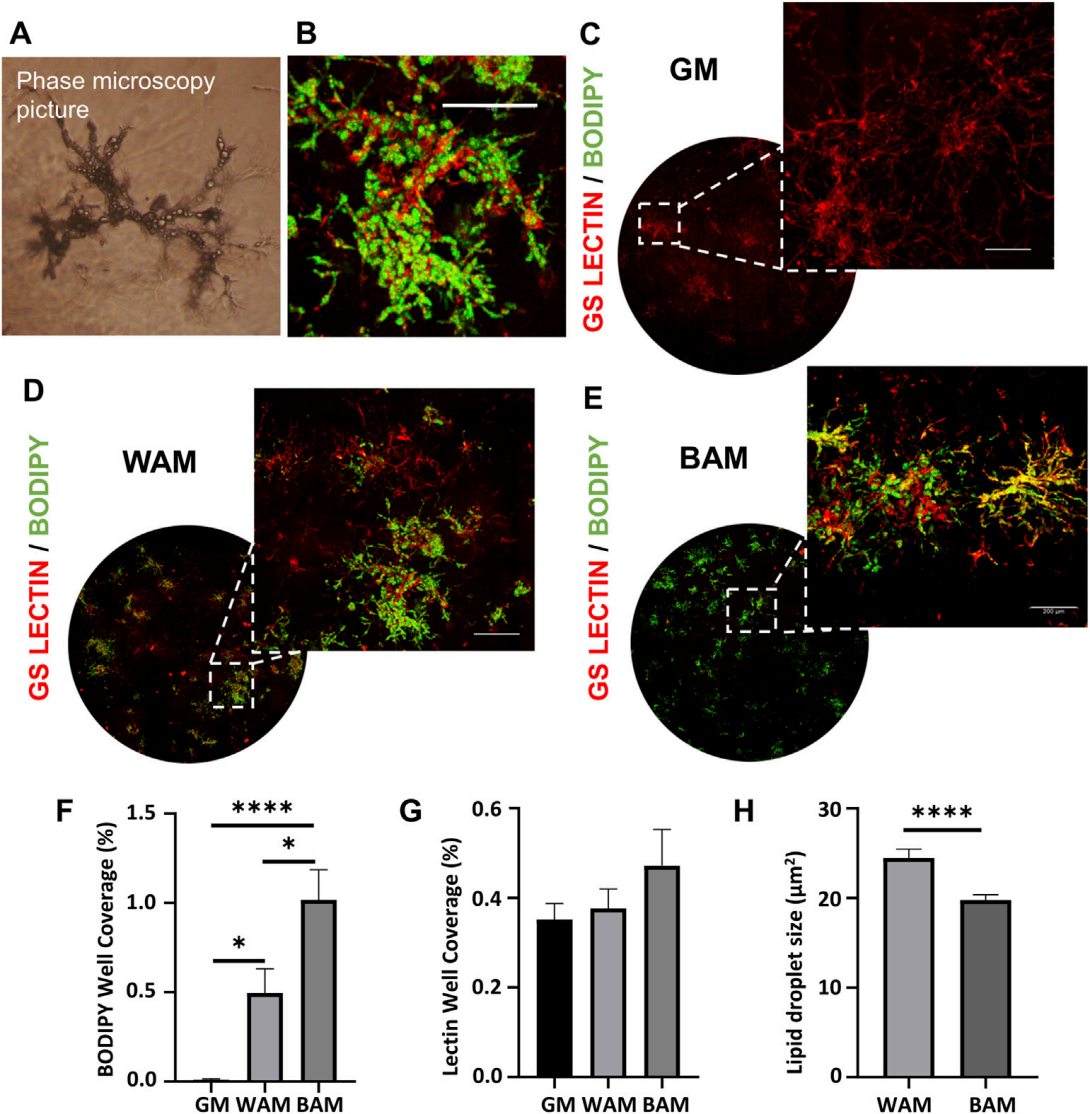
Immunofluorescence analysis of human microvascular fragments (hMVFs) beige adipogenic differentiation after 14 days. **(A)** Representative phase microscopy image of hMVFs after differentiation. **(B)** Higher magnification image of stained hMVFs (scale bar = 12 μm). **(C–E)** Representative confocal images of hMVF grown in fibrin scaffolds and stained with GS-Lectin I (red) to visualize vascular network formation and BODIPY (green) to identify the presence of lipid droplets. The images on the leftmost side display an overview of the distribution of the hMVFs in the entire well (hydrogel), and the corresponding insets on the right were included to visualize more clearly the presence of adipocytes and their interaction with the vessels (scale bars = 200 μm). **(F, G)** Quantitative analysis of lipid and vessel formation as determined with BODIPY or lectin accumulation, respectively. **(H)** Quantification of the lipid droplet size. Results are reported as mean ± standard error (*n* = 6). * = *p*< 0.05, ** = *p*< 0.01, *** = *p*< 0.001, and **** = *p*< 0.0001.

Human MVFs were suspended in fibrin gels and grown for 14 days under either GM, WAM, or BAM conditions. After 14 days of exposure to either adipogenic media (WAM or BAM), hMVFs exhibited lipid loading (Figure 1A). Under GM conditions, a network of lectin-positive cells was present, and staining for lipids was not evident (Figure 1B), while under WAM and BAM, staining of lipids with Bodipy was evident (Figures 1B,C). Quantitatively, both WAM and BAM groups showed a statistically significant higher lipid level than GM (*p* = 0.03 and *p* < 0.0001, respectively). The percentage well coverage of lipids (BODIPY staining) was two-fold greater in BAM than in WAM (Figure 1D, 1.02 ± 0.47% *vs*. 0.49 ± 0.38%). There was no difference in lectin staining (percentage of well coverage) between groups (Figure 1E). The mean size of lipid vacuoles was also compared between WAM and BAM cells. The lipids size was significantly greater in WAM than BAM (Figure 1F; *p* < 0.0001), which is consistent with the multiple smaller lipid droplets found in the cytoplasm of brown adipocytes.

**FIGURE 2:**
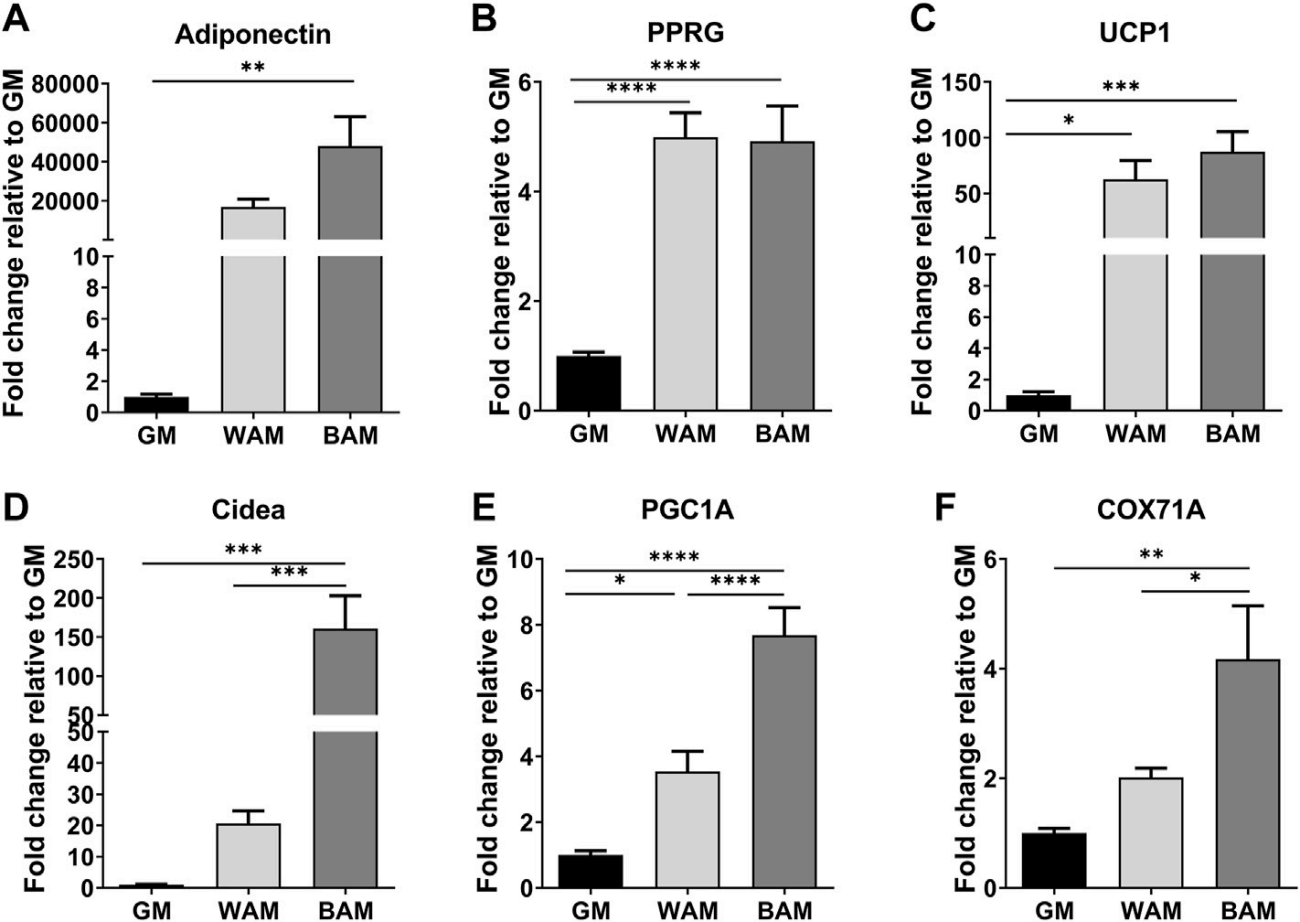
Gene expressions of adipogenic and thermogenic markers. **(A,B)** Fold expression of the adipogenic genes, adiponectin, and peroxisome proliferator-activated receptor gamma (PPRG). **(C–F)** Fold expression of several thermogenic genes, uncoupling protein 1 (UCP1), cell death-inducing DNA fragmentation factor alpha-like effector A (Cidea), peroxisome proliferator-activated receptor gamma coactivator 1-alpha (PGC-1α), and cytochrome C oxidase subunit VII a polypeptide 1 (COX71A). Results are reported as mean ± standard error (*n* = 4). * = *p*< 0.05, ** = *p*< 0.01, *** = *p*< 0.001, and **** = *p*< 0.0001.

The expression of adipogenic and thermogenic markers was analyzed using RT-qPCR to confirm the adipogenic differentiation of cells in the hMVFs (Figure 2). Genes associated with adipogenesis, including adiponectin and peroxisome proliferator-activated receptor gamma (PPRG), were greater in WAM and BAM groups than controls (GM, Figures 2A,B). Adiponectin, a protein expressed primarily in mature adipocytes, was higher in BAM than WAM (4.8 × 10^4^ in BAM vs. 1.7 × 10^4^ in WAM fold difference with respect to GM). There were no significant differences in PPRG expression levels in adipogenic conditions. The thermogenic characteristics of the systems were examined by evaluating the expression of uncoupling protein 1 (UCP1), cell death-inducing DNA fragmentation factor alpha-like effector A (Cidea) peroxisome proliferator-activated receptor gamma coactivator 1-alpha (PGC-1α), and cytochrome C oxidase subunit VIIa polypeptide 1 (COX71A) (Figures 2C–F). The expression of UCP1, a mitochondrial protein involved in thermogenic respiration, was significantly increased in BAM compared to GM. There was not a significant difference in the UCP1 expression between BAM and WAM, possibly due to the presence of forskolin in the WAM differentiation media. The expressions of Cidea (regulator of the thermogenic function in brown/beige fat), PGC-1α (transcriptional coactivator that regulates the genes involved in energy metabolism), and COX7A1 (a component of the mitochondrial respiratory chain) were significantly higher in BAM than WAM and GM, consistent with their increased expression in brown and beige adipose tissues.

### Functional Assessment of Engineered Adipose Tissues

**FIGURE 3:**
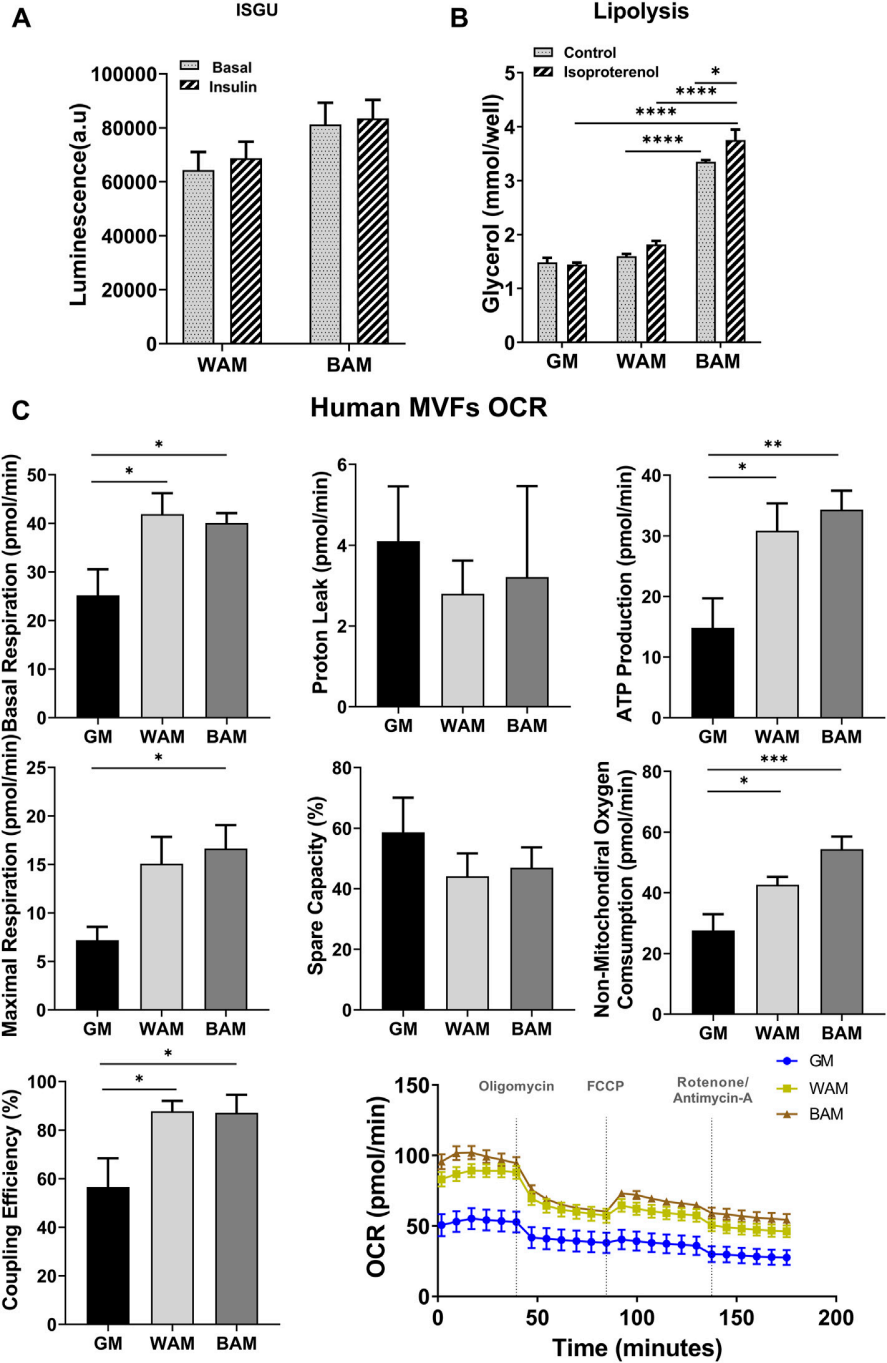
Functional analysis of beige adipose tissue formation in direct adipogenic culture of human microvascular fragments (hMVFs). hMVFs were directly exposed to white (WAM) or beige (BAM) adipogenic media for 14 days. At the end of the 14 days, **(A)** insulin-stimulated glucose uptake (ISGU) ± insulin, to stimulate glucose uptake, and **(B)** lipolysis ± isoproterenol, to stimulate lipolysis were measured. **(C)** Oxygen consumption rate (OCR) trace was determined using a Seahorse XF96 Analyzer among the different groups. Basal respiration, proton leak, maximal respiration, ATP production, non-mitochondrial oxygen consumption, coupling efficiency, and spare capacity were calculated. Results are reported as mean ± standard error of two experimental replicates (*n* = 4). * = *p*< 0.05, ** = *p*< 0.01, *** = *p*< 0.001, and **** = *p*< 0.0001.

Glucose uptake and lipolysis are expected to be higher in beige adipose microtissues. Differences in glucose uptake were assessed between WAM and BAM under basal conditions and after insulin stimulation (Figure 3A). Glucose uptake by beige microtissues was over 20% greater than white microtissues in basal conditions (6.9 × 10^4^ ± 1.2 × 10^4^ a.u. in WAM *vs*. 8.3 × 10^4^ ± 1.5 × 10^4^ a.u. in BAM) and ∼ 20% greater with insulin stimulation (6.4 × 10^4^ ± 1.5 × 10^4^ a.u. in WAM *vs*. 8.1 × 10^4^ ± 1.8 × 10^4^ a.u. in BAM) (Figure 3A). Lipolysis was assessed based on glycerol released both under basal conditions and in response to isoproterenol, a cAMP analog. Under basal conditions, media from BAM tissues contained significantly higher glycerol levels than WAM and GM (Figure 3B; *p* < 0.0001). There were differences between GM and WAM groups. BAM conditions resulted in tissues that released higher levels of glycerol relative to the control, following stimulation with isoproterenol (*p* = 0.045). There was no difference in glycerol levels between control and isoproterenol stimulated hMVF groups grown in WAM or GM (Figure 3B).

The oxygen consumption rate (OCR) was used as a measure of metabolic activity and mitochondrial respiration in the engineered microtissues. OCR was measured using standard methods with sequential exposure to oligomycin (inhibits ATP synthase), FCCP (stimulates maximal respiration), and rotenone/antimycin A (inhibits ETC and enables the calculation of non-mitochondrial respiration) over the course of 3 h (Figure 3C). Beige and white microtissues formed from MVFs exhibited significantly higher basal respiration rates relative to the GM controls (*p* < 0.05; Figure 3C). Proton leak, a measure of uncoupled respiration, was not different between groups. Maximum respiration was highest in BAM (16.63 ± 8.1 pmol/min), which was slightly greater than WAM (15.1 ± 9.2 pmol/min) and significantly greater than GM (*p* = 0.03; 7.2 ± 4.1 pmol/min). ATP production was significantly greater in BAM and WAM than GM (*p* = 0.0082 and *p* = 0.023, respectively) with no differences between BAM and WAM. The spare capacity, a measure of the ability of cells to achieve maximum respiration, was not different across groups, while non-mitochondrial oxygen consumption was greater in BAM and WAM than GM (*p* = 0.0002 and *p* = 0.036, respectively). BAM non-mitochondrial oxygen consumption was slightly higher than WAM (54.36 ± 14.34 *vs*. 42.60 ± 8.8). Sources of non-mitochondrial oxygen consumption include cell-surface oxygen consumption from electron transport at the membrane and enzymatic ROS production, which has been reported to support cells highly active in glycolysis (Muller et al., 2019).

## Discussion

Activating or increasing the amount of beige adipose tissue in an individual may improve metabolic signatures, including glucose levels, cholesterol, triglycerides, inflammation, weight gain/loss, insulin sensitivity, and glucose tolerance (Payab et al., 2021). This physiologic impact suggests that beige adipose tissues are a potential therapeutic option for the treatment of obesity and metabolic diseases (Harms and Seale, 2013). However, our understanding of beige adipose tissue development in humans is limited. In this study, we showed that human microvascular fragments, isolated from subcutaneous adipose tissue depots harvested from adults using standard minimally invasive procedures, can be used to generate adipose microtissues that recapitulate genetic and functional characteristics of beige adipose tissue. It is important to highlight that although aging is characterized by an increase in adiposity and a decline in BAT and UCP1 expressions (Zoico et al., 2019), we were able to obtain functional beige microtissues from MVFs isolated from patients all over 50 years of age.

Adipose tissue-derived MVFs consist of small segments of arterioles, venules, and capillaries (Laschke et al., 2021) and have been explored as an avenue for enhancing vascularization in engineered tissues (Laschke and Menger, 2015; Frueh et al., 2017a; Acosta et al., 2020). Although MVFs do contain the fundamental cells of microvasculature, endothelial cells, and pericytes, they also contain a variety of perivascular cells that influence tissue formation (Mcdaniel et al., 2014; Acosta et al., 2020). Specifically, isolated MVFs contain macrophages (23.6%), T-cells (0.8%), progenitor cells (11.4%), myeloid cells (5.1%), hematopoietic cells (26.7%), pericytes (28.2%), and endothelial cells (21%) (Advances Solutions, 2022). The progenitor cells include white and beige adipocyte precursors that are known to inhabit a distinct perivascular niche in the vasculature of adipose tissues. In this study, histological results demonstrated that after exposure to white and beige adipogenic media, cells present in hMVFs were able to load lipids and form adipocytes. There were more lipids under beige conditions relative to white adipogenic media. However, lipid droplets were smaller in size in BAM conditions, consistent with known features of beige adipocytes (Ikeda et al., 2018). hMVFs increased the expression of adipogenic genes upon exposure to adipogenic conditions. Interestingly, thermogenic genes, including UCP1, Cidea, PGC1α, and COX7A1, were also upregulated, following exposure to both white and beige media conditions. While the highest expression was in conditions optimized for beige adipocyte formation, it appeared that both conditions resulted in thermogenic adipose tissues that exhibited uncoupled respiration*.* This is likely due to the presence of forskolin in the WAM media. Generally, isobutylmethylxanthine (IBMX) is used as a cAMP agonist that accelerates the white adipogenic differentiation process (Petersen et al., 2008; Jia et al., 2012). However, previous studies have shown that IBMX has a detrimental effect on vessel formation when attempting to engineer vascularized adipose tissue (Yang et al., 2020). Based on these results and preliminary studies, IBMX was removed from the media and replaced with forskolin in order to preserve the ability of MVFs to maintain their vascular structure. Regardless, these data verify that hMVFs contain cells that can be induced to express markers associated with beige adipocytes.

The metabolic function of beige adipose tissues is essential for any potential therapeutic outcomes in the treatment of diabetes and obesity. Successful beige/brown adipose tissue transplantation in animal models has shown improvements in glucose metabolism and insulin sensitivity, as well as reductions in body mass and decreased adiposity (Blumenfeld et al., 2018; White et al., 2019). Hallmarks of beige/brown adipose tissues are enhanced lipid metabolism, increased glucose uptake, and mitochondrial respiration. The engineered beige adipose tissue exhibited higher baseline lipolysis relative to hMVFs exposed to white and control media conditions, and lipolysis was increased in response to the hormone isoproterenol. These results are consistent with the increase in cytosolic lipolysis that occurs in brown adipocytes (Cannon and Nedergaard, 2004). Although it did not reach a statistical difference, results from this study showed that BAT generated with hMVFs had a slight increase in basal and insulin-stimulated glucose uptake compared to WAT. Finally, for a more detailed analysis of the metabolic function of the tissue, mitochondrial bioenergetics were examined with OCR. BAM and WAM microtissues formed from hMVFs exhibited significantly higher basal respiration rates relative to the GM controls. Basal respiration is usually controlled by ATP turnover and partly by substrate oxidation and proton leak. The potential of treated cells to increase expenditure is illustrated by examining maximal respiration which was slightly greater in BAM than WAM and significantly greater than in GM. ATP production, coupling efficiency, and non-mitochondrial oxygen consumption were significantly higher in BAM and WAM than GM. Sources of non-mitochondrial oxygen consumption include cell-surface oxygen consumption from electron transport at the membrane and enzymatic ROS production which has been reported to support cells highly active in glycolysis (Muller et al., 2019). Some of the metabolic metrics were not different between BAM and WAM, possibly due to the requirement of external stimuli for beige cells to achieve maximum respiration (Brand and Nicholls, 2011) or the presence of forskolin in WAM media. As described earlier, forskolin (the cyclic AMP activator) in the media of both BAM and WAM adipocytes induced a beige-like phenotype. Future studies will focus on determining a media formulation that allows us to obtain a non-thermogenic adipose tissue. More importantly, overall, our results demonstrate increased mitochondrial respiration in hMVFs, following 3D culture supporting our hypothesis that hMVFs contain cells that can be induced to express markers and a phenotype consistent with beige adipose tissue.

We have shown that functional human beige adipose tissue can be engineered using a single cell source (hMVFs). These data demonstrate the feasibility of engineering an autologous tissue that could be used in a transplantation-based anti-obesity approach. The generation of bioengineered tissues is dependent on identifying an easily accessible and sufficient source of progenitor cells. One of the most common sources of cells for the generation of adipocytes has been preadipocytes from the stromal vascular fraction of WAT (Cawthorn et al., 2012). Although a purified population of preadipocytes or adipocytes may enable the formation of beige adipocytes, it may not be optimal for building a functional tissue. The vascular system is essential for supporting the function and formation of beige adipose tissue, and MVFs have been shown to enable rapid vascularization of tissues post-transplantation (Spater et al., 2021). For example, interactions between the adipocytes and the vascular niche may be important for browning (Bi et al., 2014), particularly through cell–cell interactions, cytokines, and growth factors such as Il-33 (Brestoff et al., 2015) or VEGF-A (Sun et al., 2012). Thus, the use of human MVFs (just 1 cell source) derived from human WAT may enable rapid vascularization of engineering beige adipose tissue. In addition, hMVFs provide the distinct advantage of studying progenitor cells in their native perivascular niche, serving as a better system for approximating *in vivo* behaviors. This approach could enable the personalized design of adipose tissue for the study of the beige adipose formation and to screen therapeutic interventions.

## Data Availability

The raw data supporting the conclusions of this article will be made available by the authors, without undue reservation.
